# The oncogeriatric transition in Morocco: health-system challenges and strategic priorities in an aging society

**DOI:** 10.3389/fpubh.2026.1834694

**Published:** 2026-05-25

**Authors:** Oussama Sabri, Halima Abahssain, Wydad Nadir, Sihame Lkhoyaali, Saber Boutayeb, Hind Mrabti, Hassan Errihani

**Affiliations:** 1 Oncology and Medical Specialties Department, Valenciennes General Hospital, Valenciennes, France; 2 Medical Oncology Department, National Institute of Oncology, Rabat, Morocco; 3 Equipe de Recherche en Oncologie Translationnelle (EROT), Faculty of Medicine and Pharmacy, University Mohammed V in Rabat, Rabat, Morocco; 4 Mohammed VI Center for Research and Innovation (CM6RI), Rabat, Morocco; 5 Mohammed VI Faculty of Medicine, Mohammed VI University of Sciences and Health (UM6SS), Casablanca, Morocco

**Keywords:** cancer epidemiology, frailty, geriatric oncology, health systems, middle-income countries, Morocco, population aging

## Abstract

**Background:**

Morocco is experiencing rapid demographic aging alongside a rising cancer burden, creating structural challenges for the care of older adults with cancer. This review synthesizes current evidence on geriatric oncology in Morocco and proposes a conceptual framework to guide system-level adaptation.

**Methods:**

We conducted a structured narrative review of peer-reviewed publications, population-based registry data, national demographic reports, and policy documents published between 2000 and 2025 (last search: January 2026). Evidence was synthesized qualitatively and organized into six predefined analytical domains: (1) demographic transition, (2) cancer epidemiology, (3) health system organization, (4) access to care, (5) workforce capacity, and (6) geriatric assessment and clinical practice. These domains are applied consistently as the organizing framework across the Results sections and are explicitly mapped onto the WHO Health System Building Blocks and the Four-Phase Oncogeriatric Transition framework in the Discussion.

**Results:**

In 2024, adults aged ≥60 years accounted for 13.8% of Morocco's population, while individuals aged ≥65 years represented approximately 8%, with projections indicating a marked increase by 2050. Population-based registries report age-standardized cancer incidence rates around 120–137 per 100,000. Available cohorts indicate high vulnerability prevalence (e.g., >80% abnormal G8 in some series), substantial metastatic presentation at diagnosis, limited geriatric workforce capacity, and a strong urban concentration of oncology services. Structured geriatric assessment is not yet consistently implemented in routine oncology care.

**Conclusion:**

These findings suggest that Morocco is entering an oncogeriatric transition characterized by a growing mismatch between demographic acceleration and geriatric-integrated oncology capacity. We propose a Four-Phase Oncogeriatric Transition framework to conceptualize this evolution and inform policy, workforce planning, and phased implementation strategies. Early integration of geriatric assessment, registry adaptation, and multidisciplinary coordination will be essential to ensure equitable, age-adapted cancer care in an aging society.

## 1 Introduction

Morocco is undergoing a marked demographic transition characterized by a sustained increase in the proportion of older adults. Although national demographic and administrative reports commonly define “older persons” using a ≥60-year threshold, this review adopts ≥65 years as its primary analytical definition to align with major geriatric oncology standards and international clinical guidelines. When data are only available using the ≥60-year definition, they are reported explicitly as such and interpreted within their national policy framework, to avoid analytical conflation. According to the 2024 General Population and Housing Census (RGPH 2024) and international population estimates, approximately 8.1% of Morocco's population, equivalent to ~3.0–3.2 million people, were aged ≥65 years in 2024, while adults aged ≥60 years accounted for ~13.8% (around five million individuals) (1, 2).

United Nations World Population Prospects (WPP) 2022 (medium-variant scenario) projects that the number of adults aged ≥65 years in Morocco will **nearly double by 2050**, reflecting continued fertility decline and sustained gains in life expectancy (2, 3). This demographic shift is expected to amplify the burden of chronic conditions and multimorbidity, placing increasing pressure on healthcare delivery, social protection mechanisms, and the organization of long-term and supportive care. Adapting cancer care to the complex needs of older adults will therefore become a strategic health-system priority.

In parallel, cancer incidence in Morocco has increased steadily over recent decades. Population-based cancer registries in Casablanca and Rabat report age-standardized incidence rates of approximately **120–140 per 100,000 inhabitants**, with breast, lung, and colorectal cancers among the most frequent sites (4, 5). As in other settings, cancer is predominantly a disease of aging; consequently, the proportion of older adults among incident cases is expected to increase further as life expectancy rises. However, Morocco still lacks robust national, age-stratified cancer data specifically focused on older adults, with evidence largely limited to a small number of population-based registries and single-center series.

Geriatric oncology has emerged internationally as a discipline at the interface of oncology and geriatrics, aiming to individualize cancer care by incorporating comorbidities, functional status, cognition, nutrition, psychosocial context, and patient preferences into treatment decision-making. International evidence and society recommendations (including SIOG) indicate that geriatric assessment can improve therapeutic selection, reduce toxicity, and optimize quality of life in older adults with cancer (6). In Morocco, however, geriatric oncology remains at an early stage of development. Data on older Moroccan patients with cancer are scarce, and the first multicenter study describing their sociodemographic and clinical characteristics highlighted a population with strong social ties but substantial economic vulnerability (7).

Taken together, these trends indicate an urgent need to clarify how the Moroccan health system currently responds to the specific needs of older adults with cancer. The convergence of demographic aging and rising cancer burden operates through interconnected biological, social, and health system mechanisms—each amplifying the others and collectively generating structural pressure on oncology care systems that were not designed with older, frailer populations in mind. Existing evidence points to gaps in cancer registration, geographic and financial access to oncology services, and availability of geriatric expertise; yet these issues have not been comprehensively synthesized through an oncogeriatric, health-system lens. This narrative review addresses this gap within a dual analytical framework—the WHO Health System Building Blocks and a novel Four-Phase Oncogeriatric Transition model—to summarize available evidence, identify critical gaps, and propose pragmatic staged priorities for service development and research in a middle-income North African context.

## 2 Methods

This narrative review was conducted using a structured and transparent methodology to identify and synthesize available evidence on geriatric oncology in Morocco. While not designed as a systematic review or meta-analysis, the review followed a predefined search and selection strategy to enhance reproducibility and methodological rigor.

### 2.1 Search strategy

A structured literature search was performed in PubMed/MEDLINE and Google Scholar for articles published between January 2000 and December 2025, with the last search conducted in January 2026. The strategy combined controlled vocabulary (MeSH terms) and free-text keywords related to aging, cancer, geriatric oncology, and Morocco, including: “Morocco,” “cancer,” “oncology,” “elderly,” “older adults,” “aged,” “geriatrics,” “geriatric oncology,” “frailty,” “access to care,” and “palliative care.” Boolean combinations included: [“Morocco” AND cancer AND (elderly OR “older adults” OR geriatrics OR frailty)]. Reference lists of included sources were also screened to identify additional relevant publications.

#### 2.1.1 Eligibility criteria

We included observational studies, population-based registry reports, multicenter analyses, reviews, doctoral theses, and official policy documents reporting Moroccan data on cancer epidemiology, clinical characteristics, treatment patterns, outcomes, healthcare organization, or access to care in adults aged ≥65 years. The ≥65-year threshold was selected to align with international geriatric oncology standards and major clinical guidelines. When Moroccan sources used a ≥60-year definition, data were extracted as reported and interpreted within the corresponding national demographic and policy framework. When geriatric-specific studies were unavailable, broader oncology publications with relevant age-stratified analyses or health-system insights were retained. We excluded case reports without epidemiological relevance, conference abstracts lacking accessible full data, and publications not providing Moroccan information.

#### 2.1.2 Gray literature

Given the limited volume of peer-reviewed geriatric oncology research in Morocco, gray literature was included to capture the health-system and policy context. Sources included documents from the Ministry of Health and Social Protection, the National Cancer Prevention and Control Plan, population-based cancer registries (Casablanca and Rabat), the High Commission for Planning, and the Economic, Social and Environmental Council.

### 2.2 Study selection and synthesis

#### 2.2.1 Overview of included literature

To enhance transparency and allow the reader to assess the evidential basis of the narrative synthesis, we provide a high-level characterization of the body of literature identified and retained. The search strategy yielded a corpus of sources spanning peer-reviewed publications, gray literature, and policy documents. Retained sources comprised: population-based cancer registry reports (Casablanca and Rabat registries); nationally representative demographic reports and projections (RGPH 2024, UN WPP 2022, High Commission for Planning); peer-reviewed observational studies and cohort analyses focused on older Moroccan adults with cancer, including the first multicenter cross-sectional study and single-center series; gray literature from governmental and intergovernmental bodies (Ministry of Health, Economic Social and Environmental Council, WHO, Human Rights Watch); and international guidelines and consensus statements from SIOG, ASCO, and ESMO, retained for benchmarking purposes rather than as primary evidence on the Moroccan context.

Studies were included when they provided Moroccan data relevant to at least one predefined analytical domain. Sources reporting exclusively on non-Moroccan populations were retained only when they provided the international benchmarking or conceptual context necessary to interpret Moroccan data. Conference abstracts were included when they represented the sole available source of site-specific or tumor-specific data on older Moroccan patients, and are explicitly identified as such throughout the manuscript.

A deliberate decision was made to exclude case reports without epidemiological relevance, conference abstracts for which full data were not accessible, and publications not providing Moroccan information. Several analytical domains yielded no Moroccan peer-reviewed publications, a finding that is itself reported as a critical evidence gap rather than treated as a reason for exclusion of the domain from the synthesis.

The resulting body of evidence is heterogeneous in design and quality, as expected in a field at an early stage of development in a middle-income setting. This heterogeneity directly informed both the choice of narrative synthesis methodology and the structure of the proposed Four-Phase Oncogeriatric Transition framework, which was designed to accommodate and reflect the variable granularity of available evidence across system domains.

### 2.3 Quality considerations

Consistent with the narrative design, no formal quality appraisal tool (e.g., Newcastle-Ottawa Scale, GRADE) was applied—an absence explicitly acknowledged as a methodological limitation. In lieu of formal assessment, a structured hierarchy of interpretative weight was applied consistently: highest weight was assigned to population-based registry data and nationally representative demographic reports; intermediate weight to multicenter observational studies; and cautious interpretation to single-center series, given their susceptibility to referral and urban selection bias. Gray literature was retained for governance and policy insights but treated as contextual rather than primary clinical evidence. Conference abstracts were included only where they represented the sole available Moroccan source for a given domain and are explicitly flagged as such throughout the manuscript. Heterogeneity across sources—in design, population age definitions (≥60 vs. ≥65 years), institutional context, and data collection period—was managed through domain-based qualitative synthesis rather than cross-study quantitative comparison, and is acknowledged as a constraint on the strength of conclusions drawn.

#### 2.3.1 Analytical framework

To ensure analytical coherence and provide an explicit theoretical grounding for this review, evidence was synthesized within a dual conceptual framework combining two complementary models. First, the World Health Organization (WHO) Health System Building Blocks framework—encompassing service delivery, health workforce, health information systems, access to essential medicines, financing, and leadership/governance—was used as a structural lens to organize the assessment of Morocco's oncogeriatric capacity across system dimensions [WHO REF]. This framework was selected because it provides a validated, internationally recognized architecture for evaluating health system performance in resource-constrained settings and maps directly onto the structural gaps identified in the Moroccan oncogeriatric landscape: workforce shortages, geographic concentration of services, limited health information on older adults with cancer, financing barriers, and governance gaps in age-adapted care policy.

Second, we propose the Four-Phase Oncogeriatric Transition framework as an original complementary contribution, designed to capture the dynamic, temporal dimension of health system adaptation to demographic aging—a dimension not addressed by the static Building Blocks model. Where the WHO framework describes what components a health system possesses or lacks at a given moment, the Four-Phase model describes how systems evolve over time as the convergence of demographic aging and rising cancer burden generates progressive structural pressure. Together, these frameworks provide both a cross-sectional structural diagnosis and a longitudinal trajectory model, enabling the synthesis of heterogeneous evidence—demographic, epidemiological, clinical, and organizational—into a coherent analytical narrative applicable to Morocco and transferable to other middle-income settings undergoing compressed demographic transitions.

The following sections present findings organized across three analytical domains—demographic and epidemiological context, health system organization and access to care, and geriatric assessment and clinical practice—structured within the dual WHO Building Blocks and Four-Phase Oncogeriatric Transition framework described above.

## 3 Results

### 3.1 Results: demographic and epidemiological context

This section addresses the first two analytical domains: demographic transition and cancer epidemiology.

#### 3.1.1 Aging of the Moroccan population

For methodological consistency, epidemiological interpretations in this review prioritize the ≥65-year threshold. Data reported using a ≥60-year definition are included for contextual relevance but are not considered interchangeable with ≥65-year estimates.

Morocco has entered a phase of accelerated population aging, driven by sustained improvements in life expectancy and declining fertility. According to the 2024 General Population and Housing Census, approximately 5.0 million individuals aged ≥60 years accounted for 13.8% of the population in 2024, representing a substantial increase compared with earlier decades (1).

Using the ≥65-year threshold applied throughout this review, approximately 8% of the Moroccan population—corresponding to ~3.0–3.2 million individuals—was aged ≥65 years in 2024 (2). Projections suggest that the population aged ≥60 years may approach 10 million by 2050, potentially representing nearly one quarter of the national population (8). This implies a major expansion of the ≥65-year population over the coming decades, with direct implications for oncology demand, multimorbidity management, and long-term supportive care capacity.

Population aging in Morocco is also shaped by social and economic vulnerability. Women constitute a slight majority among older adults due to higher life expectancy and are disproportionately affected by widowhood, limited pension coverage, and poverty^1^. Moreover, gender-related disparities intersect with aging to influence health trajectories, access to care, and cancer outcomes. Older women may face delayed diagnosis due to financial dependency, lower health literacy, and sociocultural barriers to healthcare access, particularly in rural settings. At the same time, they represent a substantial proportion of patients affected by high-incidence cancers such as breast cancer (9).

Conversely, gender norms may also shape health-seeking behaviors, patterns of comorbidity, and reliance on informal caregiving among older men. These gendered dynamics interact with broader structural determinants—including geographic disparities, health system organization, and access to social protection—and may contribute to inequalities in cancer presentation, treatment pathways, and outcomes (10). Despite these implications, sex-disaggregated data on older adults with cancer remain limited in Morocco, highlighting an important gap for future research and policy development.

#### 3.1.2 Cancer burden and its relationship to aging

Cancer is a growing public health challenge in Morocco, and its burden is closely linked to demographic aging. Population-based cancer registries in Casablanca and Rabat report age-standardized incidence rates of approximately **120–140 per 100,000**, with breast, lung, and colorectal cancers among the most frequent sites (4, 5). Registry analyses indicate that incidence rises sharply with age and that a substantial share of cases occurs after the age of 60, consistent with established age-specific cancer patterns (4).

**Figure 1:**
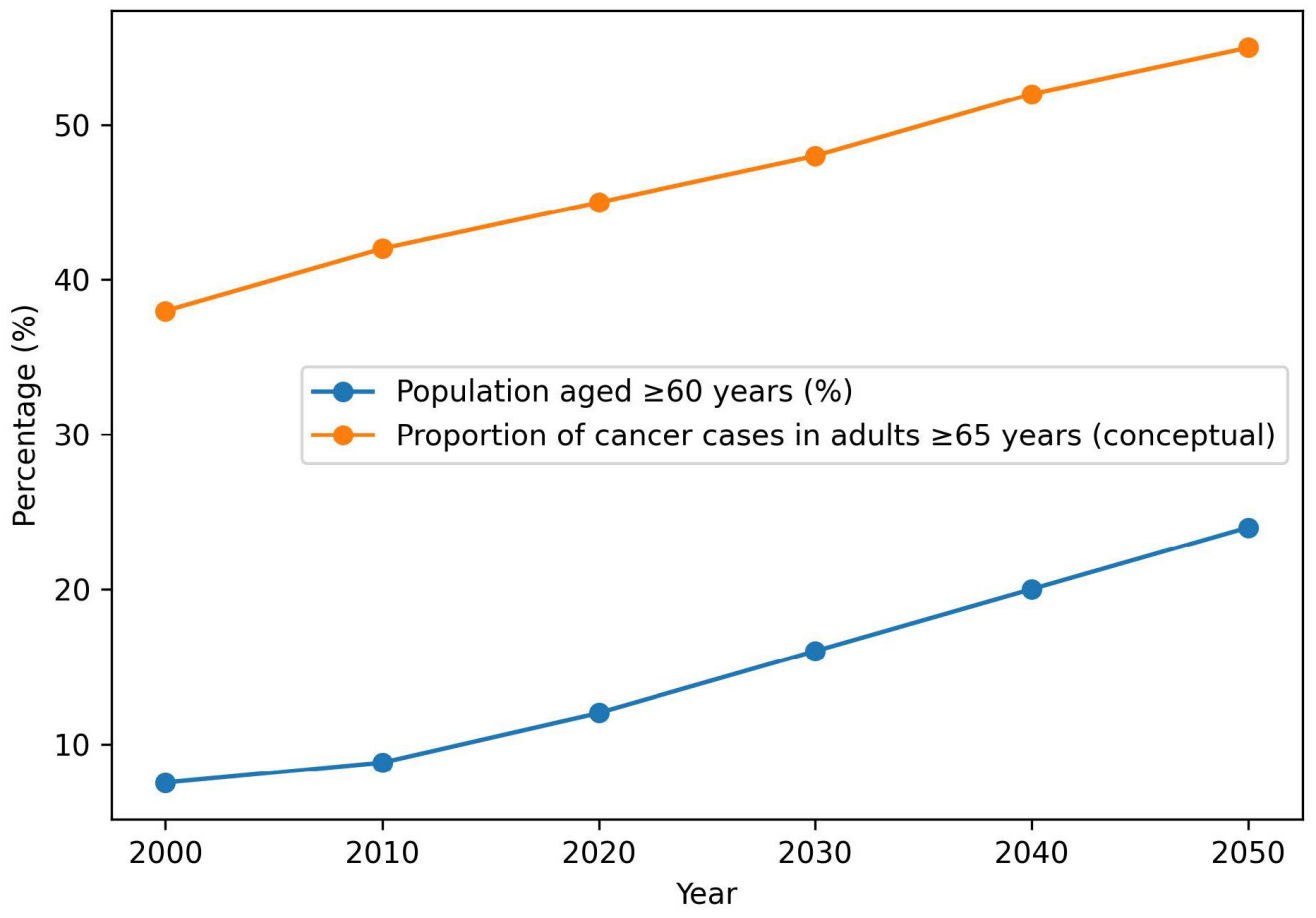
Conceptual convergence between demographic aging and cancer burden in Morocco (2000–2050).

Nevertheless, detailed national evidence focusing specifically on older adults with cancer remains limited. The first multicenter Moroccan study dedicated to older cancer patients, conducted across nine oncology departments, reported frequent economic vulnerability, low educational attainment, and comorbidity, alongside relatively strong family support (7). The same study underscored that geriatric assessment and structured geriatric oncology services are not yet widely implemented in routine care (7). Overall, the convergence of accelerated population aging and increasing cancer incidence (Figure 1) supports the urgency of developing age-adapted oncology strategies in Morocco.

The projected proportion of adults aged ≥60 years is derived from national demographic data and medium-variant projections from the High Commission for Planning and UN WPP (8, 11). The curve representing the proportion of cancer cases occurring in adults aged ≥65 years reflects established age-specific incidence patterns from Moroccan population-based registries and global surveillance data (GLOBOCAN) (4, 12).

In the absence of official age-stratified national projections for older adults, this curve is presented as a conceptual illustration under an assumption of relatively stable age-specific incidence rates; it is not an official forecast.

### 3.2 Results: health system organization and access to care

This section addresses the third and fourth analytical domains: health system organization and access to care.

#### 3.2.1 Cancer care infrastructure

Cancer care in Morocco is delivered through a mixed network of university hospitals, regional oncology centers, and private facilities; however, major geographic and resource disparities persist. Comprehensive oncology services (surgery, systemic therapy, and radiotherapy) remain concentrated in large urban hubs such as Casablanca, Rabat, Fès, and Marrakech, while many rural and remote areas face limited local access to specialized care. National Cancer Prevention and Control Plans, supported by the Lalla Salma Foundation, have contributed to the creation and upgrading of regional oncology centers and to the development of multidisciplinary pathways, yet persistent gaps remain in equipment availability, maintenance, and trained personnel—particularly outside major cities (13, 14).

Palliative care illustrates these structural constraints. Human Rights Watch reported in 2016 that structured palliative care was available in only a small number of institutions—mainly in Casablanca and Rabat—alongside very low national opioid consumption and regulatory and educational barriers to adequate pain relief *(“Pain Tears Me Apart” 2016) (“Pain Tears Me Apart” 2016)*. More recent national profiles and WHO-oriented briefs suggest gradual progress, including the opening of additional hospital-based palliative care units (e.g., Agadir and Béni Mellal) and the development of home-based palliative care initiatives during the COVID-19 pandemic to maintain continuity of care^2^, (15, 16). Nevertheless, palliative care coverage remains limited, and many patients with advanced cancer still lack access to comprehensive symptom control and end-of-life support (Table 1).

**Table 1:** The table summarizes regional cancer epidemiology and oncology resources, highlighting the strong concentration of specialist capacity along the Casablanca–Rabat axis (including the majority of oncologists and the near-totality of geriatric services), despite these regions representing only a portion of the national older adult population.

Region	Population (M, 2024)	% ≥ 60 years	Cancer registry (ASR/100k)	Main oncology centers	Estimated oncologists/radiotherapist (~370 Total)	Estimated geriatricians (< 10 residents)
Casablanca-Settat	7.6	14.1%	137.3 (2008–12)	CHU Ibn Rochd, Center Mohammed VI, Private Clinics (Badr, Littoral)	~150–160 (43%)	~3–4 residents
Rabat-Salé-Kénitra	4.6	14.0%	124.8 (2006–08)	INO Rabat (National Center), CHU Ibn Sina, Military Hospital	~140–150 (40%)	~2–3 residents + 2 public units
Fès-Meknès	4.2	14.5%	Registry in dev.	CHU Hassan II Fès, CRO Meknès	~20–25	1
Marrakech-Safi	4.5	13.6%	68.0 (2012–19)	CHU Mohammed VI Marrakech, CRO Safi (planned)	~20–25	1
Oriental	2.4	16.1%	Hospital-based (CIR 8.9)	CHU Mohammed VI Oujda, CRO Al Hoceima	~10–15	0
Béni Mellal-Khénifra	2.5	15.2%	No PBCR	Proximity Center Béni Mellal	~5–10	0
Tanger-Tétouan-Al Hoceima	3.8	11.8%	No PBCR	CHU Mohammed VI Tanger	~10–15	0
Other Regions (Souss-Massa, Southern, etc.)	7.4	4.8%−14.1%	No PBCRs	CRO Agadir, CRO Laâyoune	~10–20	0
TOTAL Morocco	36.8–37.0	13.8% (~5M)	137.3 (National Est.)	31 centers (Public/Private)	~370 specialists	< 10 residents

ASR/ASIR, Age-Standardized (Incidence) Rate; 100k, Per 100,000 inhabitants; CHU, University Hospital Center; INOR, National Institute of Oncology; CRO, Regional Oncology Center; RGPH, General Population and Housing Census; PBCR, Population-Based Cancer Registry.

For older adults, these infrastructure limitations are compounded by age-related vulnerabilities. Long travel distances, fragmented referral pathways, limited transport options, and the lack of systematically organized social support may disproportionately affect older patients with mobility limitations, multimorbidity, or cognitive impairment. Delays in diagnostic work-up and treatment initiation—reported in several Moroccan series—may be particularly detrimental in frail older adults whose functional reserve can deteriorate rapidly. Overall, while Moroccan oncology capacity has expanded over the past two decades, it remains insufficiently adapted to the clinical complexity of an aging population (7, 13, 17).

#### 3.2.2 Access to cancer care for older adults

Access to cancer care in Morocco remains constrained by financial, geographic, and sociocultural barriers that may disproportionately affect older adults. A recent review estimated diagnostic delays ranging from **160 to 219 days** and highlighted key obstacles including travel distance, transport constraints, out-of-pocket expenditures, limited insurance coverage, and intermittent drug availability in public facilities (13). These delays are amplified by geographic barriers, as patients may travel a median distance of **118 km** to reach specialized oncology centers, generating additional costs for transportation and accommodation (18). High illiteracy rates among older adults (reported at **71.6%**) and economic vulnerability (with **50.4%** reporting no income source) may further limit their ability to navigate administrative pathways, including increasingly digitalized systems (1, 19).

While the National Cancer Prevention and Control Plan and the generalization of Mandatory Health Insurance (AMO) represent important steps toward universal coverage, specialized geriatric resources remain extremely limited^3^. Morocco faces a severe shortage of geriatricians and has only two public hospital structures dedicated specifically to older adults^4^ (Table 1).

### 3.3 Results: geriatric assessment, workforce, and clinical practice

This section addresses the fifth and sixth analytical domains: workforce capacity and geriatric assessment and clinical practice.

#### 3.3.1 Workforce capacity and geriatric expertise

Morocco faces a substantial healthcare workforce gap, with a reported medical density of 6.7 per 10,000 inhabitants, below the WHO-recommended benchmark of 15.3 (13) (see text footnote 4). Within this constrained system, geriatric expertise is particularly limited, as there is currently no national diploma for specialization in geriatrics (7). Consequently, only a small number of medical residents are formally training in geriatrics nationwide, and although university diplomas in geriatrics are held by some general practitioners, this capacity remains insufficient relative to the pace of population aging (see text footnote 4).

These workforce limitations are further compounded by geographic concentration: a large majority of oncology specialists are located along the Casablanca–Rabat corridor, and the only two public hospital units dedicated to older adults are situated within the Rabat–Salé–Kénitra region (13). In response, national policy actors, including the Economic, Social and Environmental Council, have called for the progressive structuring of a “care economy,” with ambitious workforce expansion targets by 2035 (see text footnote 4).

Critically, no established benchmark exists for the number of oncogeriatric specialists per population, reflecting the early and still-emerging nature of this field globally. In practice, dedicated oncogeriatric practitioners remain extremely scarce even in high-income settings: care for older adults with cancer is most commonly delivered either by oncologists without formal geriatric training or by geriatricians without oncology-specific expertise (20). This structural gap has been documented even in high-resource tertiary care settings (21).

Morocco is no exception to this global pattern (22). Consequently, the management of older adults with cancer is predominantly ensured by general oncologists, with limited systematic integration of geriatric principles into routine clinical decision-making (7).

Several European countries report general geriatrician densities in the range of approximately 24 to 50 per million inhabitants (23); the near-absence of equivalent capacity in Morocco therefore reflects both a general geriatric deficit and a specific oncogeriatric gap that mirrors challenges documented across many health systems worldwide.

#### 3.3.2 Use of geriatric assessment tools

Geriatric assessment (GA) is internationally recognized as a cornerstone of geriatric oncology, enabling individualized treatment planning by evaluating functional status, comorbidity, cognition, nutrition, polypharmacy, psychosocial context, and support systems. Evidence supports GA-based approaches to identify frailty, anticipate toxicity, and inform shared decision-making (24).

Current international guidelines—including those from the American Society of Clinical Oncology (ASCO) and the European Society for Medical Oncology (ESMO)—recommend systematic GA for older adults receiving systemic anticancer therapy (6, 25). Recommended tools span two complementary functions: screening instruments designed to identify patients requiring full geriatric evaluation, such as the G8 and the Vulnerable Elders Survey-13 (VES-13), and toxicity prediction models, including the Cancer and Aging Research Group (CARG) score and the Chemotherapy Risk Assessment Scale for High-Age Patients (CRASH), which estimate the risk of severe treatment-related toxicity and support individualized therapeutic decisions (26–29).

Despite strong guideline endorsement, implementation of GA in routine oncology practice remains inconsistent globally. Key barriers include time constraints within standard oncology consultations, limited availability of trained personnel, insufficient institutional support, and lack of familiarity with geriatric tools among oncologists (30, 31). Consequently, GA is most often applied selectively rather than systematically, even in high-income settings with established oncology infrastructure (32). These real-world implementation gaps underscore that the challenge is not merely one of tool availability, but of organizational integration—a distinction that is particularly relevant in resource-limited contexts where structural barriers are compounded by workforce shortages and limited training pathways.

In Morocco, GA implementation is emerging as a clinical and policy priority, particularly within the National Strategy for Older Adults' Health (2024–2030), which aims to institutionalize geriatric evaluations in specialized consultations (19).

Clinicians and researchers have used several international tools, including the Fried frailty phenotype and the G8 screening tool for oncology vulnerability detection (33). Complementary measures include ADL/IADL for functional independence, the Mini Nutritional Assessment (MNA), and the Geriatric Depression Scale (GDS) (19). To our knowledge, predictive toxicity tools such as CARG and CRASH have not yet been formally evaluated in the Moroccan oncology setting, representing an important evidence gap given the high prevalence of frailty and comorbidity in available cohorts.

A central challenge is cultural and educational adaptation: cognitive and functional tests requiring calculations or written/drawing tasks may be inappropriate in the context of high illiteracy among older adults. In response, efforts are underway to develop a Moroccan-adapted CGA integrating locally relevant indicators, including functional proxies grounded in everyday and religious practices (e.g., ablutions and prayer) (7).

Single-center experience has provided early feasibility evidence. A doctoral thesis from the National Institute of Oncology in Rabat explored implementation of a composite geriatric assessment approach in older cancer patients, suggesting that simplified tools can be operationalized in busy oncology workflows to stratify vulnerability and inform treatment intensity (34). These findings support the rationale for routine structured screening in Moroccan oncology settings and highlight the need for prospective validation studies adapted to the local context.

### 3.4 Treatment patterns in older adults

Available evidence on treatment patterns in older Moroccan adults with cancer remains limited in scope but consistently highlights several structural features. Data from the first multicenter Moroccan study on older cancer patients indicate that treatment strategies were predominantly delivered with palliative intent (65.2% vs. 34.8% curative), a pattern likely driven by late-stage presentation: nearly 44.2% of older patients were metastatic at diagnosis (7). Chemotherapy was the dominant therapeutic modality, used in 88.6% of cases, while clinicians reported adapting treatment intensity in approximately 19.2% of patients to account for advanced age and comorbidity burden (7).

Beyond these aggregate figures, emerging site-specific data provide more granular insight into treatment patterns across major tumor types. For colorectal cancer, a retrospective study at Hassan II University Hospital in Fès identified 62 patients aged ≥70 years over a three-year period, representing 27.5% of all colorectal cancer cases at that institution (35). Notably, 37% were metastatic at diagnosis, with a mean diagnostic delay of 7.3 months—consistent with the broader pattern of late-stage presentation documented across tumor types in Morocco. Treatment-related toxicity was observed across all chemotherapy regimens, and 11% of patients received no systemic treatment due to comorbidities or deteriorated performance status, underscoring the clinical complexity of this population and the authors' own conclusion that geriatric assessment is essential in the management of older patients with colorectal cancer (35).

In a cohort of older women with metastatic breast cancer, **50%** received chemotherapy as first-line therapy, despite a predominance of luminal subtypes that might otherwise favor endocrine-based approaches, highlighting potential practice variability and access constraints (36). Clinicians reported adapting approximately **19.2%** of treatments to account for advanced age and comorbidity burden (7).

Clinical complexity is further increased by a high prevalence of vulnerability, with **83.4%** screening positive using the G8 tool in one series (19).

For prostate cancer, a prospective single-center study conducted in Morocco evaluated 86 patients aged ≥70 years with a G8 score ≤ 14 using a comprehensive pre-therapeutic oncogeriatric assessment (OGA) incorporating comorbidity burden (CIRS-G), nutritional status (MNA), functional autonomy (ADL/IADL), mobility, muscle strength, cognition (Mini-Cog), and psychosocial status (37). Following OGA, patients were stratified into robust (32%), vulnerable (40%), and frail (28%) categories, with therapeutic strategies adapted accordingly: exclusive palliative care for frail patients, active surveillance or radiotherapy fractionation for a subset of vulnerable patients, and standard treatment for robust patients. Severe toxicity (grade ≥3) occurred in 21.6% of patients overall and was significantly more frequent in the vulnerable group compared to the robust group (33.5% vs. 8.2%, *p* = 0.03). At 12 months, overall survival was 81.2% and progression-free survival 76.4%; in multivariate analysis, vulnerability status independently predicted disease progression (HR = 2.4, 95% CI 1.2–4.8, *p* = 0.02), alongside metastatic stage and PSA >20 ng/mL (37).

A further structural challenge concerns the evidence base underlying treatment decisions in older adults globally. Older patients have historically been underrepresented in oncology clinical trials, resulting in limited age-specific efficacy and safety data across most tumor types. Consequently, systemic therapy regimens and dosing strategies are frequently extrapolated from trials conducted predominantly in younger, fitter populations—a practice that may inadequately account for pharmacokinetic alterations, reduced functional reserve, higher comorbidity burden, and increased toxicity risk characteristic of older adults. This evidence gap is particularly consequential in resource-limited settings such as Morocco, where limited capacity for treatment individualization may amplify the risks associated with protocol extrapolation in the absence of locally validated algorithms.

The findings synthesized across these three domains are discussed below in relation to international evidence, structural mechanisms, and the proposed Four-Phase Oncogeriatric Transition framework.

## 4 Discussion

The following discussion integrates findings across the six predefined analytical domains—demographic transition, cancer epidemiology, health system organization, access to care, workforce capacity, and geriatric assessment and clinical practice—within the dual WHO Health System Building Blocks and Four-Phase Oncogeriatric Transition framework, to identify structural mechanisms, draw international comparisons, and propose staged priorities for system adaptation.

Morocco is entering a structural period in which demographic aging and the rising burden of cancer increasingly converge, mirroring patterns observed across many low- and middle-income countries (LMICs). According to the United Nations WPP 2022, the proportion of adults aged ≥65 years is projected to increase substantially across North Africa, driven by sustained gains in life expectancy and declining fertility (38).

In parallel, global cancer estimates indicate that demographic change—particularly population aging—is a central driver of projected increases in cancer incidence worldwide, with especially pronounced effects in transitioning economies (39). In this context, aging functions as a structural multiplier of cancer burden, intensifying demand for complex oncology care alongside supportive, rehabilitative, and palliative services.

The convergence of demographic aging and rising cancer burden documented in this review is not coincidental but mechanistically grounded across three interdependent levels. At the **biological level**, aging is associated with the progressive accumulation of somatic mutations, telomere attrition, immunosenescence, and chronic low-grade systemic inflammation—a process termed inflammageing—all of which increase cancer susceptibility while simultaneously altering pharmacokinetics, reducing treatment tolerance, and impairing recovery from oncological interventions (40, 41). These biological processes explain why cancer incidence rises sharply with age and why older adults with cancer represent a clinically distinct population requiring individualized therapeutic approaches rather than straightforward extrapolation from younger patient data. At the **social level**, population aging in Morocco unfolds against a backdrop of substantial economic vulnerability, high illiteracy rates among older adults, and evolving household structures in which rapid urbanization and internal migration may progressively weaken traditional intergenerational solidarity^5^. These social determinants compound clinical complexity, amplify barriers to care access, reduce treatment adherence, and may accelerate functional decline—particularly in the absence of formal social protection mechanisms adapted to the needs of older adults with cancer (42). At the **health system level**, the structural mismatch between accelerating demographic aging and an oncology infrastructure historically calibrated for younger, fitter populations generates progressive strain across all dimensions of system performance—from workforce capacity and service organization to health information systems, medicines access, and governance frameworks (21, 43, 44). It is precisely this three-level convergence that the proposed Four-Phase Oncogeriatric Transition framework seeks to conceptualize and that the WHO Health System Building Blocks model helps to operationalize diagnostically (45).

**Figure 2:**
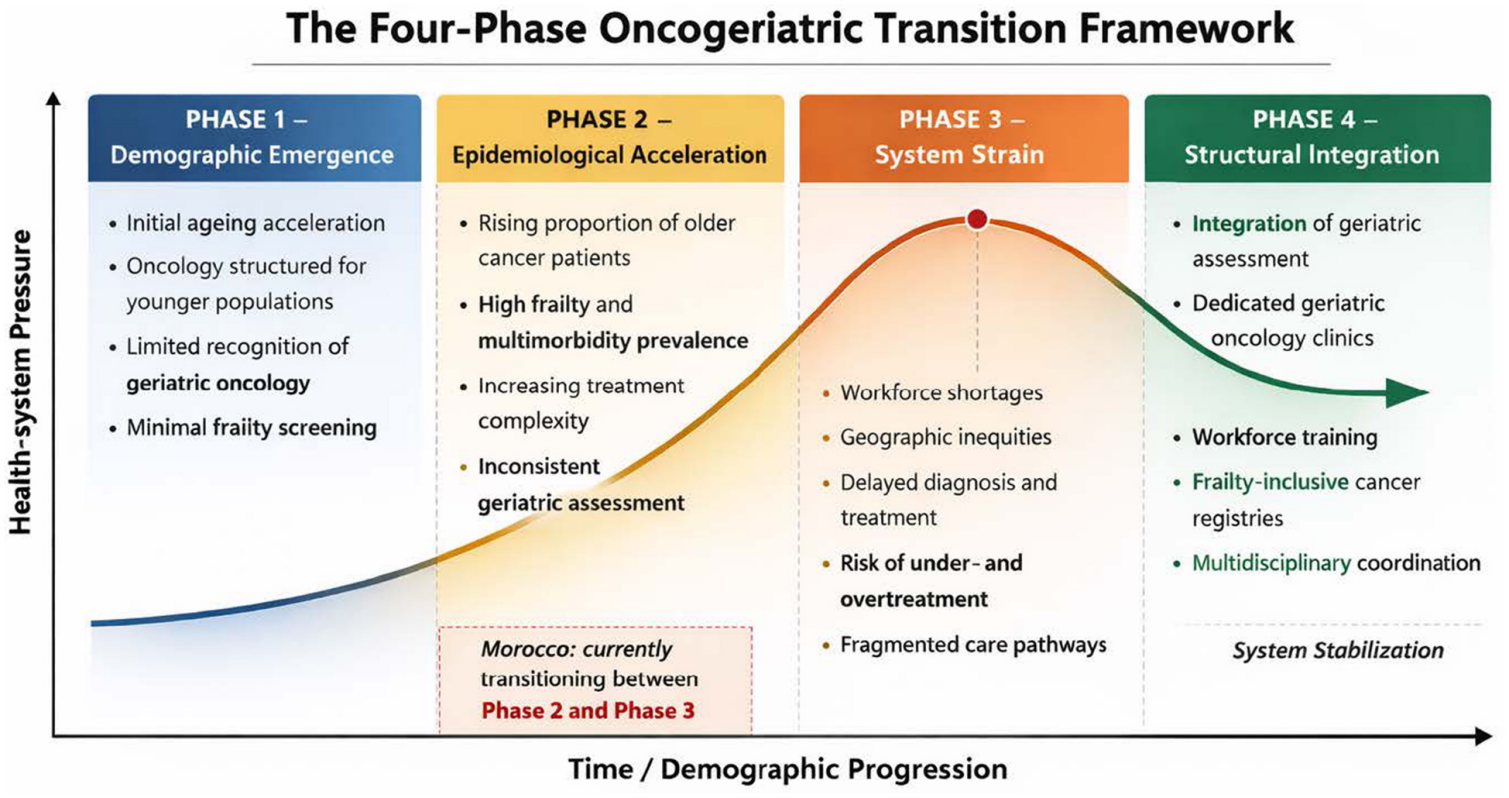
The Four-Phase Oncogeriatric Transition Framework. The model illustrates the structural evolution of health systems as demographic aging converges with increasing cancer burden. Health-system pressure rises as the proportion of older adults with cancer increases in the absence of geriatric-integrated oncology services (Phases 1–3). Structural integration of geriatric assessment, workforce adaptation, and coordinated care pathways enables system stabilization (Phase 4). Morocco currently appears to be transitioning between the epidemiological acceleration and system strain phases.

These dynamics suggest that Morocco is entering what may be conceptualized as an **Oncogeriatric Transition**—a phase in which accelerated population aging converges with rising cancer burden **without proportional expansion of geriatric-informed oncology capacity**, increasing health-system strain and the risk of age-inappropriate cancer care. We propose a four-phase model to conceptualize this evolution in middle-income settings undergoing compressed demographic change: (Figure 2).

**Phase 1–Demographic Emergence:** Aging accelerates, but oncology systems remain structured around younger/middle-aged populations; geriatric oncology is not formally integrated.
**Phase 2–Epidemiological Acceleration:** The proportion of older adults with cancer rises; vulnerability and multimorbidity become common, yet geriatric assessment remains inconsistently implemented.
**Phase 3–System Strain:** A mismatch emerges between clinical complexity and system capacity. Workforce shortages, limited geriatric expertise, geographic inequities, and fragmented referrals contribute to delays, heterogeneous treatment patterns, and risks of under- and overtreatment.
**Phase 4–Structural Integration:** Systems respond by integrating geriatric assessment into oncology pathways, developing dedicated services, expanding training, adapting registries to incorporate geriatric variables, and strengthening coordination across oncology, primary care, and palliative care.

Based on the evidence synthesized, Morocco appears to be positioned between **Phase 2 and Phase 3**, characterized by rapid growth of the older cancer population and emerging structural pressure without full institutional integration of geriatric oncology services. This framework may also be relevant to other North African and LMIC contexts facing similarly rapid demographic shifts.

The reviewed evidence highlights important gaps at the oncology–geriatrics interface. The high prevalence of vulnerability observed in Moroccan cohorts is consistent with international estimates reporting vulnerability in a substantial proportion of older adults with cancer, depending on tools and populations (46).

International guidelines (SIOG, ASCO) recommend routine geriatric assessment to support treatment individualization and reduce toxicity risk (6, 25). However, in Morocco, implementation remains inconsistent, largely due to workforce shortages, limited training pathways, and organizational constraints (47).

Workforce limitations constitute a central structural barrier. Global geriatrician shortages are well documented even in high-income settings, and the gap is often magnified in lower-resource systems (48). In Morocco, the extreme concentration of geriatric services within a narrow geographic corridor further amplifies inequities in access to age-adapted oncology care.

Organizational and access constraints also disproportionately affect older adults. Diagnostic and treatment delays exceeding 160–200 days reported in Moroccan series are particularly concerning given evidence linking prolonged time-to-treatment initiation with poorer outcomes across several malignancies (13, 49).

Geographic concentration of specialist services is a common pattern in LMICs and contributes to travel-related financial burden, delayed presentation, and fragmented follow-up (49, 50). These barriers may be especially harmful for older patients with reduced mobility, multimorbidity, limited income, and dependence on informal caregiving.

Beyond these observed gaps, a more explicit examination of underlying structural and policy-level determinants is required to understand how and why these constraints emerge. Several interrelated mechanisms can be identified across health governance, workforce development, and access to essential resources.

First, national health policy and governance frameworks have prioritized expansion of oncology infrastructure through successive National Cancer Prevention and Control Plans (PNPCC), leading to increased availability of regional oncology centers^6^, (51). However, these strategies have historically focused on disease-specific capacity expansion (e.g., radiotherapy units, chemotherapy access) rather than on the integration of geriatric principles into oncology care pathways. As a result, structural components such as comprehensive geriatric assessment, multidisciplinary coordination with geriatricians, and age-adapted care models remain insufficiently institutionalized (25).

Second, human resource development represents a critical bottleneck. The absence of a formal national specialization pathway in geriatrics, combined with limited training in geriatric oncology within oncology curricula, constrains the system's ability to operationalize guideline-recommended practices (7, 52). This results in a cascade effect whereby limited geriatric expertise reduces the implementation of geriatric assessment, which in turn contributes to non-individualized treatment strategies, increased risk of toxicity, and potential under- or overtreatment in older adults (53).

Third, policies affecting access to medicines, supportive care, and diagnostic technologies further shape clinical practice. While national insurance reforms and public oncology programs have improved access to anticancer therapies, disparities persist in supportive care availability, including palliative care, nutritional support, and rehabilitation services—components that are particularly critical for frail older patients (54, 55). These gaps reinforce a model of care that remains predominantly tumor-centered rather than patient-centered.

Importantly, these structural factors do not operate in isolation but interact with broader social determinants, including poverty, illiteracy, and geographic inequities, thereby amplifying vulnerability among older adults (56). This interaction helps explain why delays in diagnosis, advanced-stage presentation, and limited treatment adaptation are recurrent findings across Moroccan studies.

Palliative care integration remains another critical challenge. The Lancet Commission on Palliative Care and Pain Relief has emphasized that limited access to essential pain medicines and end-of-life services in LMICs contributes to avoidable suffering in advanced cancer (49, 50, 57). Although Morocco has initiated expansions in palliative structures and home-based initiatives, coverage remains incomplete, and systematic integration into oncology pathways remains variable.

These findings should also be interpreted in light of gender-related disparities, which may influence access to care, vulnerability, and treatment trajectories among older adults, although such dimensions remain insufficiently captured in available Moroccan data. Future research and cancer registries should integrate gender as a key stratification variable to better inform equitable care strategies.

International comparisons are presented below across three consistent analytical dimensions—oncogeriatric workforce development, GA implementation in routine practice, and existence of context-specific adaptation strategies—with explicit acknowledgment of structural differences limiting direct transferability.

**Egypt**, the most comparable North African middle-income setting, shares Morocco's rapid demographic transition and concentrated oncology infrastructure. An implementation study at Ain Shams University Hospital (*n* = 117, older adults receiving chemotherapy) found 73.5% G8-positive and GA-guided treatment modification in ~20% of cases (58). Like Morocco, Egypt lacks a formal oncogeriatric specialty; implementation remains institution-driven rather than policy-mandated. Morocco is now generating comparable prospective evidence, as illustrated by the recently presented prostate cancer OGA cohort (37).

**Brazil**, an upper-middle-income comparator with a longer oncogeriatric tradition, demonstrates what Phase 4 integration can look like: a decade-long experience at a public university cancer center documented systematic G8 screening, embedded multidisciplinary geriatric oncology services, and measurable treatment individualization benefits (59). Key structural differences from Morocco—larger distributed geriatric workforce, established academic infrastructure, universal health system platform—limit direct transferability but confirm that scalable models are achievable in middle-income contexts.

**European high-income settings** illustrate that GA implementation gaps are not resource-dependent alone: scoping reviews consistently document inconsistent integration despite guideline endorsement and workforce availability (31). This finding disaggregates the implementation problem into resource availability and organizational integration—both of which Morocco must address simultaneously on its trajectory toward Phase 4.

Across all three contexts, vulnerability prevalence in older adults with cancer ranges from 60–85%, GA-guided treatment modification occurs in 15–25% of cases when systematically applied, and workforce/organizational barriers dominate over tool availability—a convergence that strengthens the cross-contextual applicability of the proposed framework.

Based on this synthesis, several staged priorities emerge for Morocco. First, pilot geriatric oncology services could be established within major oncology centers (e.g., INOR Rabat; university hospitals in Casablanca and Marrakech) using validated screening tools (e.g., G8) to identify high-risk older adults and trigger more comprehensive assessment and supportive interventions, consistent with international recommendations (6).

Second, training should be strengthened through structured geriatric oncology modules in residency programs and continuing education, as educational capacity is a prerequisite for sustainable implementation (6).

Third, cancer registries and health information systems should progressively incorporate age-stratified variables relevant to older adults (frailty, comorbidity, functional status, and toxicity), improving both clinical planning and health-system forecasting. Finally, stronger coordination between oncology, primary care, and palliative care services is required to ensure continuity for frail older adults, particularly in underserved regions; early integration of supportive and palliative interventions is essential to reduce avoidable morbidity and suffering (57).

This review has several limitations. As a structured narrative synthesis, it integrates heterogeneous sources, including population-based registry reports, single-center studies, and policy documents, which may introduce variability and potential urban referral bias. The absence of a nationwide population-based cancer registry further limits the ability to draw fully representative national-level inferences, while prospective, outcome-driven geriatric oncology studies remain scarce in Morocco.

Although international evidence supports the role of geriatric assessment (GA) in reducing treatment-related toxicity and improving outcomes, comparable validation studies in Moroccan settings are still lacking. In addition, no formal quality appraisal tool was applied to individual sources, reflecting the heterogeneity of the evidence base and the narrative design; this limitation is explicitly acknowledged.

Cross-country comparisons, while structured around consistent analytical domains, remain constrained by differences in data availability, study design, and health system organization, and should therefore be interpreted as illustrative rather than systematic. Finally, the proposed Four-Phase Oncogeriatric Transition framework, although grounded in the synthesized evidence, remains a conceptual model that requires prospective empirical validation across diverse low- and middle-income country settings.

Despite these limitations, the convergence of demographic, epidemiological, and workforce indicators supports the relevance of this framework to inform strategic planning and staged health system adaptation in the context of population aging and rising cancer burden.

## 5 Conclusion

Morocco is approaching a pivotal period in its healthcare trajectory as rapid demographic aging converges with a growing cancer burden. This dual transition will substantially increase the number of older adults requiring complex, individualized oncologic and supportive care. Current evidence suggests a structural mismatch between rising needs and limited geriatric-integrated oncology capacity, driven by workforce shortages, geographic inequities, limited systematic vulnerability assessment, and incomplete registry integration. The proposed Four-Phase Oncogeriatric Transition framework provides a practical conceptual lens to anticipate system strain and support staged planning. Prioritizing geriatric assessment integration, workforce training, registry adaptation, and coordinated supportive care pathways offers an opportunity to proactively shape equitable, evidence-based cancer care for an aging society and may inform other middle-income settings facing similarly compressed demographic transitions.

## Footnotes

1 Available online at: https://www.cese.ma/media/2020/10/Rapport-Les-personnes-%C3%A2g%C3%A9es-au-Maroc.pdf (Accessed February 28, 2026).

2 Available online at: https://www.hrw.org/sites/default/files/report_pdf/morocco0216_web.pdf (Accessed February 11, 2026).

3 Available online at: https://www.contrelecancer.ma/site_media/uploaded_files/PNPCC_2020_2029.pdf (Accessed February 28, 2026).

4 Available online at: https://www.cese.ma/media/2025/10/Rapport-annuel-2024-web-1.pdf (Accessed February 28, 2026).

5 Available online at: https://www.un.org/development/desa/pd/content/world-population-ageing-2023.

6 Available online at: https://www.contrelecancer.ma/site_media/uploaded_files/PNPCC_2020_2029.pdf (Accessed April 11, 2026).
